# Horizon scan of DNA-based methods for quality control and monitoring of herbal preparations

**DOI:** 10.3389/fphar.2023.1179099

**Published:** 2023-05-03

**Authors:** Ancuța Cristina Raclariu-Manolică, Quentin Mauvisseau, Hugo J. de Boer

**Affiliations:** ^1^ Stejarul Research Centre for Biological Sciences, National Institute of Research and Development for Biological Sciences, Piatra Neamț, Romania; ^2^ Natural History Museum, University of Oslo, Oslo, Norway

**Keywords:** DNA barcoding, innovation, herbal product, quality control, genomic barcoding

## Abstract

Herbal medicines and preparations are widely used in healthcare systems globally, but concerns remain about their quality and safety. New herbal products are constantly being introduced to the market under varying regulatory frameworks, with no global consensus on their definition or characterization. These biologically active mixtures are sold through complex globalized value chains, which create concerns around contamination and profit-driven adulteration. Industry, academia, and regulatory bodies must collaborate to develop innovative strategies for the identification and authentication of botanicals and their preparations to ensure quality control. High-throughput sequencing (HTS) has significantly improved our understanding of the total species diversity within DNA mixtures. The standard concept of DNA barcoding has evolved over the last two decades to encompass genomic data more broadly. Recent research in DNA metabarcoding has focused on developing methods for quantifying herbal product ingredients, yielding meaningful results in a regulatory framework. Techniques, such as loop-mediated isothermal amplification (LAMP), DNA barcode-based Recombinase Polymerase Amplification (BAR-RPA), DNA barcoding coupled with High-Resolution Melting (Bar-HRM), and microfluidics-based methods, offer more affordable tests for the detection of target species. While target capture sequencing and genome skimming are considerably increasing the species identification resolution in challenging plant clades, ddPCR enables the quantification of DNA in samples and could be used to detect intended and unwanted ingredients in herbal medicines. Here, we explore the latest advances in emerging DNA-based technologies and the opportunities they provide as taxa detection tools for evaluating the safety and quality of dietary supplements and herbal medicines.

## Introduction

Herbal products play a crucial role in healthcare systems worldwide, but their quality and safety raise concerns (Zhang et al., 2012; Ekor, 2014). Over the past few years, there has been a steady rise in the use of commercial herbal products (Garcia-Alvarez et al., 2014; Smith et al., 2021), and a diverse range of new dietary supplements and herbal medicines continue to enter the market under very heterogenous regulatory frameworks (Thakkar et al., 2020). Globally, there is no consensus regarding the definition and characterization of these herbal products that often come from a great variety of local and traditional practices. Numerous commercial terms, including herbal or botanical drugs, traditional or herbal medicines, natural health products, and dietary or food supplements (Alostad et al., 2020), are being used to describe these products.

Commercial herbal products are biologically active mixtures, with complex and variable contents, making it difficult to assess their quality and safety using conventional analytical methods (Zhang et al., 2012; Shipkowski et al., 2018; Heinrich et al., 2022). These products have globalized value chains, creating additional concerns such as responsible sourcing of raw plant materials and sustainable supply (Booker et al., 2012; Heinrich et al., 2019). While specific quality issues are strictly monitored for certain categories of herbal products classified as “medicines” in some jurisdictions (Thakkar et al., 2020), “supplements” typically have less rigorous premarket and post-marketing regulations, leaving room for safety issues (Posadzki et al., 2013; Heinrich, 2015; Teschke and Eickhoff, 2015; Steinhoff, 2019). Nevertheless, the research community, along with some regulatory authorities and Pharmacopoeias, is making significant contributions and proposing innovative strategies to identify and authenticate botanicals and their derived preparations, ensuring quality control (Simmler et al., 2018; Fitzgerald et al., 2019; Durazzo et al., 2021; Heinrich et al., 2022).

Due to the high demand and increasing prices for herbal preparations, supply chains for raw plant materials are sometimes unable to keep up, leading to accidental contamination or intentional adulteration for economic gain (Ichim, 2019; Ichim et al., 2020; Ichim and Booker, 2021; Gafner et al., 2023). Traditional approaches for botanical identification and quality control, including botanical taxonomy, macroscopic and microscopic examination, and phytochemical analysis to detect specific characteristics or compounds have been reviewed previously (Upton et al., 2019; Klein-Junior et al., 2021). However, as discussed by Gafner et al. (2023), fraudulent operators are aware of these identification assays and have found ways to deceive them. Unethical adulteration practices, combined with natural complexity and potential human errors, have a significant impact on the quality of botanical products (Heinrich, 2015). Furthermore, non-compliant physical labels and false online claims of herbal products pose additional safety hazards (You et al., 2022; Jordan et al., 2023).

**FIGURE 1:**
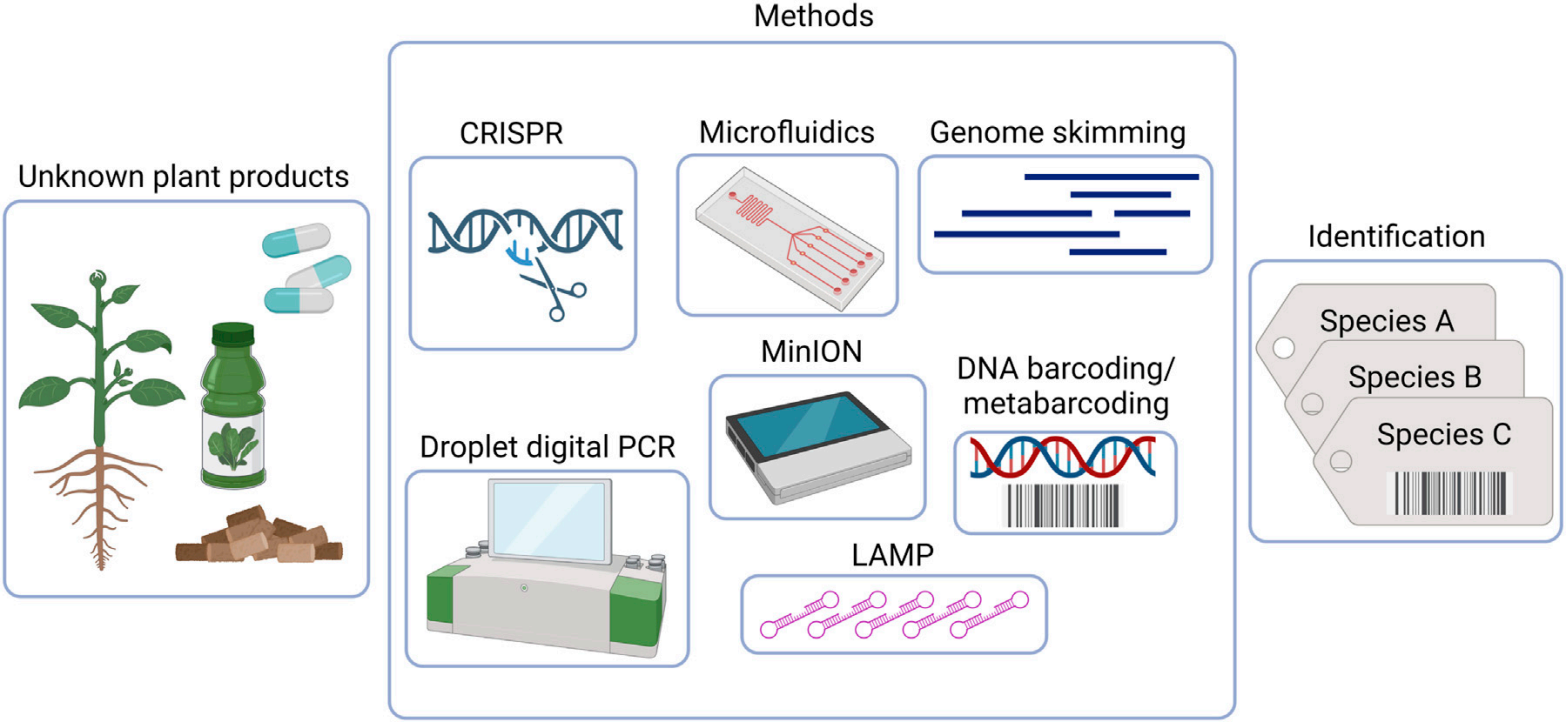
DNA-based methods for quality control and monitoring of herbal preparations. Created with BioRender.com.

Given the current circumstances, it is recommended to adopt innovative and alternative testing approaches to ensure the quality control of herbal products (Simmler et al., 2018; Thakkar et al., 2020; Abraham and Kellogg, 2021). In recent years, high-throughput sequencing (HTS) methods have revolutionized our ability to provide insights into the total species diversity in DNA mixtures. After two decades of remarkable progress, the concept of standard DNA barcoding has significantly expanded. This article discusses the latest advancements in emerging DNA barcoding-based technologies and the potential opportunities they offer as taxa detection tools for evaluating the safety and quality of dietary supplements and herbal medicines (See Figure 1 and Table 1).

**TABLE 1:** Comparison of the DNA-based methods for quality control and monitoring of herbal preparations.

	Analysis cost per sample	Universality	Species diversity assessment	Degraded DNA	PCR free	Sequencing free	Sample multiplexing	Portable	Detection of untargeted plant species in complex samples
DNA barcoding		**✓**	**✘**	**✘**	**✘**	**✘**		**✘**	
Bar-HRM					**✘**	**✓**		**✘**	
DNA metabarcoding		**✓**	**✓**		**✘**	**✘**	**✓**	**✘**	**✓**
DNA mini-barcoding				**✓**	**✘**	✓*	✓*	**✘**	
ddPCR/dPCR					**✘**	✓*	**✓**	**✘**	
LAMP					**✓**	**✓**		**✓**	
BAR-RPA					**✓**	**✓**		**✓**	
Microfluidics LAMP					**✓**	**✓**	**✓**	**✓**	
CRISPR-microfluidic array					✓*	✓*	**✓**	**✓**	
MEBarcoding					**✘**	**✘**	**✓**	**✘**	
Genome skimming	**✘**	**✓**	**✓**	**✓**	**✓**	**✘**	**✓**	**✘**	**✓**
Target capture sequencing	**✘**	**✓**	**✓**	**✓**	**✓**	**✘**	**✓**	**✘**	**✓**
MinION DNA metabarcoding		**✓**	**✓**		**✘**	**✘**	**✓**	**✓**	**✓**

Note: DNA based methods were “roughly” compared to highlight their clear benefits and/or limitations. The (**✓**) symbol highlight a clear advantage or benefit of the method, and the (**✘**) symbol highlight a clear downside or limitation. When lacking information, or when the method did not showcase a clear benefit or limitation for the given characteristic, no symbol was added. When * is indicated, the method can be applied by using more than one analytical approach and the assessment may vary accordingly.

## State-of-the-art—DNA barcoding

DNA barcoding is an identification system that uses short and standardized regions of the genome, known as “barcodes”, as advocated by Hebert et al. (2003). The concept is straightforward—when a DNA barcode is obtained from a taxonomically unknown specimen, a computational algorithm compares it against a reference database containing reference sequences with known taxonomy to identify the targeted barcode. The technique operates on the assumption that the level of intraspecific genetic divergence is lower than the interspecific genetic divergence, forming a cut-off threshold, the “barcoding gap” (Meyer and Paulay, 2005). A query sequence is considered to be distinct from a reference sequence if the difference is above this threshold (Hebert et al., 2003; Meyer and Paulay, 2005). Different loci from chloroplast and nuclear genomes (e.g., *trnH-psbA*, *rbcL*, *matK, ycf5,* ITS2) have been proposed for medicinal plant DNA barcoding (Chen et al., 2010).

The application of DNA barcoding has been a significant breakthrough for both the research community and industry, enabling it to address a range of questions that were previously impossible to answer. Standardized DNA barcoding protocols have been proposed for the identification of plant material by Pharmacopeias around the world, including the Chinese, United States, Japanese, and British Pharmacopoeias (Howard et al., 2009; Kreuzer et al., 2019; Ha et al., 2022; Wu and Shaw, 2022). The utility of DNA barcoding to detect adulteration has been shown in studies on herbal products derived from *Ginkgo biloba* L. (Little, 2014a), *Actaea racemosa* L. (Baker et al., 2012), and species belonging to the genera *Senna* and *Cassia* (Seethapathy et al., 2014), among others (Ghorbani et al., 2015; Hart et al., 2016). It has been applied also to detect common adulterants of endangered species used in herbal medicines (e.g., genus *Panax*) (Zuo et al., 2011; Wallace et al., 2012), and to identify species with a high toxic or allergenic potential present in marketed herbal products (Wallace et al., 2012; Seethapathy et al., 2014; Wang et al., 2017). While DNA barcoding is particularly useful for molecular identification and traceability of raw materials (Galimberti et al., 2013), this method is not always effective in detecting species substitution or adulteration in mixed DNA samples and with high levels of DNA degradation (Raclariu et al., 2018). DNA barcoding does not have the capacity to screen for chemical compounds, thus has no capacity to test the presence of toxic constituents or synthetic substances that may be present within the herbal products.

## DNA barcoding coupled with high-resolution melting (Bar-HRM)

Bar-HRM is a cost-effective, sequence-independent technique that rapidly and accurately authenticates crude herbal materials post-PCR. Initially developed by Jaakola et al. (2010) for the authentication of berries, it has since been extensively applied to identify herbal medicines (Sun et al., 2016). Unlike DNA barcoding which utilizes specific and standardized genomic regions as genetic barcodes to differentiate species, HRM detects sequence variations by analyzing DNA melting curves. Combined, they enable rapid and accurate species identification, particularly in complex environmental samples where morphological methods may be challenging. For instance, Kalivas et al. (2014) used Bar-HRM combined with ITS2 barcode to identify *Sideritis* species, while Song et al. (2016) discriminated *Artemisa* species and authenticated commercial products in China. Using *rbcL* Bar-HRM primers, Osathanunkul and Madesis (2019) differentiated edible and poisonous ginseng species, Osathanunkul et al. (2015) authenticated medicinal *Acanthaceae* species commonly used in Thailand and Tungphatthong et al. (2021) tested various Bar-HRM barcodes to identify *Mitragyna speciosa* (Korth.) Havil., a species prohibited in Thailand.

## DNA metabarcoding

DNA metabarcoding is a cutting-edge technique that combines the traditional concept of DNA barcoding with high-throughput sequencing (HTS), allowing for the identification of multiple taxa from complex mixtures and matrices containing DNA from different sources (Taberlet et al., 2012; Raclariu et al., 2018). In the field of herbal product authentication, DNA metabarcoding has a significant advantage over traditional DNA barcoding due to its ability to identify many species from complex mixtures and matrices at any stage of processing or production (de Boer et al., 2015; Raclariu et al., 2018). It is highly effective in assessing total species diversity in processed/finished products, in post-marketing control and pharmacovigilance, and has been successfully applied in several studies to reveal a disturbing prevalence of adulteration (Ichim, 2019). The use of undeclared plant fillers or accidental substitution materials is among the suspected causes of the high number of adulterated commercial products (Coghlan et al., 2012; Coghlan et al., 2015; Ivanova et al., 2016; Raclariu et al., 2017; Seethapathy et al., 2019). In some cases, DNA metabarcoding identified species protected by the Convention on International Trade in Endangered Species of Wild Fauna and Flora (CITES) (Coghlan et al., 2015; de Boer et al., 2017), and a multi-locus DNA metabarcoding method has been developed to detect these species (Arulandhu et al. (2017). Additionally, DNA metabarcoding allows the detection of species with high toxicity and allergenic potential in herbal products (Cheng et al., 2014; Speranskaya et al., 2018; Anthoons et al., 2021; Frigerio et al., 2021).

## Current research gaps

DNA metabarcoding is an extremely sensitive technique, capable of identifying trace amounts of contamination, such as a single grain of pollen (Polling et al., 2022). However, the quantification of abundance is complicated by a range of factors that can lead to false positive detections. These can include contamination, adulteration, amplification bias, sequencing errors, and errors in reference databases used for taxonomic assignment (Hawkins et al., 2015). To minimize the risk of false positive detections, biological and technical replicates should be used, and strict bioinformatics filtering criteria should be applied (Ficetola et al., 2015). On the other hand, degraded DNA caused by harvesting, drying, storage, transportation, and processing (Novak et al., 2007), difficulties in DNA extraction due to the presence of pharmaceutical excipients (Costa et al., 2015), poor primer fit and amplification biases (Piñol et al., 2015), stochasticity due to low DNA concentrations (Giguet-Covex et al., 2014), or incomplete reference databases may lead to false negative results. To overcome these challenges and to ensure the quality of the DNA barcoding procedure, the use of reference standards as positive controls for DNA extraction and PCR is highly recommended (Sgamma et al., 2017). Moreover, the use of a positive control library based on synthetic DNA molecules (gBlocks) has been shown to be a good strategy (Sgamma et al., 2018; Howard et al., 2020a; Howard et al., 2020b).

## Current developments and horizon scan

### DNA mini-barcoding

DNA mini-barcoding is an efficient identification method target short (≤200–300 bp) DNA regions that have the capacity to overcome some limitations of conventional DNA barcoding related to DNA fragmentation in herbal ingredients and processed products (Meusnier et al., 2008; Little, 2014b). The approach has a significant species identification potential in samples that contain degraded DNA and/or more than one species (Gao et al., 2019). Various strategies have been applied to develop specific mini-barcodes to be used for species identification in herbal products. Howard et al. (2009) proposed species-specific DNA mini-barcodes to discriminate *Hypericum perforatum* L. from other closely related *Hypericum* spp., and these are able to detect even low amounts of targeted DNA. The use of multiplex DNA metabarcoding of multiple mini-barcode loci has shown promising taxon identification potential in complex samples with heavily degraded DNA, and such approaches are applicable to monitor the illegal trade of endangered plant and animal species used in traditional medicines (Arulandhu et al., 2017). Yu et al. (2020) used specific mini-barcodes targeting chloroplast sequences combined with metabarcoding for qualitative and quantitative estimation of *Senna obtusifolia* (L.) H. S. Irwin and Barneby in processed herbal products.

### Droplet digital PCR (ddPCR) and digital PCR (dPCR)

Droplet digital PCR (ddPCR) and digital PCR (dPCR) are powerful techniques that allow to detect and quantify DNA or RNA molecules in a sample. Following the partition of a sample into thousands of nanoliter-sized droplets or partition (using either emulsion or microfluidic-based approaches), a fluorescence reading of each droplet/partition allows accurate detection and quantification of low-level target DNA, even in the presence of inhibitors in complex samples. Compared to quantitative PCR, ddPCR offers numerous benefits, including higher sensitivity and precision, as well as an absolute measure of nucleic acid concentration without the need for standard curves (Hindson et al., 2011; Pinheiro et al., 2012; Hindson et al., 2013). Fit-for-purpose approaches are increasingly used to quantify DNA targets at low levels in various applications (Pinheiro et al., 2012), with ddPCR being used to identify and quantify animal species in meat and meat products (Floren et al., 2015). ddPCR has been applied to herbal product authentication, and several studies highlighted its potential. Yu et al. (2020), Yu et al. (2022) developed assays targeting respectively *Panax ginseng* C.A.Mey. (ginseng), *Oryza sativa* L. (rice), and *Glycine max* (L.) Merr. (soybean), to enable their detection and quantification in complex mixtures, and Xu et al. (2022) detected and quantified low levels of adulterants, i.e., Mu Tong (*Akebia quinata* (Houtt.) Decne.) and *Aristolochia manshuriensis* Kom., which contain several aristolochic acids, which are known carcinogens able to cause kidney toxicity. Unlike qPCR which is sensitive to inhibition and only provides relative quantification, ddPCR enables absolute quantitative evaluations from low amounts of the target DNA even in the presence of chemical and protein contaminants or inhibitors (Techen et al., 2014; Taylor et al., 2017).

### Loop-mediated isothermal amplification (LAMP)

Loop-mediated isothermal amplification (LAMP) was first introduced as an alternative to PCR-based methods by Notomi et al. (2000) and amplifies target-specific DNA sequences under isothermal conditions without significant interference from a non-target template. Compared to PCR-based methods, LAMP is more sensitive and specific (Craw and Balachandran, 2012), does not require expensive equipment or special molecular techniques (Li et al., 2016), and can enable on-site analysis (Kogovšek et al., 2015; Sillo et al., 2018; Köppel et al., 2019). These advantages have led to the rapid adoption of LAMP in various fields, including food safety testing for foodborne pathogens (Niessen et al., 2013; Law et al., 2014; Zhong and Zhao, 2018), genetically modified organisms (Singh et al., 2019), and food allergens such as *Pistacia vera* L. (pistachio), *Glycine max* (L.) Merr. (soybean), and *Arachis hypogaea* L. (peanut) (Sheu et al., 2018; Allgöwer et al., 2020; Mao et al., 2020). Li et al. (2016) reviewed the potential of LAMP for identifying raw medicinal plant materials and proposed a practical Standard Operating Procedure (SOP) for utilizing LAMP in herbal authentication. LAMP has been used for identifying and authenticating medicinal plants such as *Curcuma longa* L., *Catharanthus roseus* (L.) G. Don., *Hedyotis diffusa* Willd., *Zingiber officinale* Roscoe, and *Taraxacum formosanum* Kitam. (Sasaki and Nagumo, 2007; Chaudhary et al., 2012; Li et al., 2013; Chaudhary and Khan, 2014; Lai et al., 2015). While Zhao et al. (2016) developed ITS2-specific LAMP primers targeting *Crocus sativus* L. (saffron) to enable simple and sensitive differentiation from adulterants, Fochi et al. (2022) developed a LAMP-based diagnostic protocol for the authentication of food supplements made from the edible fungus *Grifola frondosa* (Dicks.) Gray (“Maitake”). This was successfully tested on commercial products, demonstrating its potential for routine inspections at any level of the production chain.

### DNA barcode-based recombinase polymerase amplification (BAR-RPA)

DNA barcode-based Recombinase Polymerase Amplification (BAR-RPA) is a technique that uses recombinase, polymerase, and single-stranded binding proteins (SBB) to rapidly amplify genetic markers in as little as 15–20 min under a constant temperature of 37°C–42°C. Without the need for thermocyclers, this innovative approach can replace the traditional PCR technique’s unwinding chain process (Piepenburg et al., 2006; Lobato and O’Sullivan, 2018). Tian et al. (2017) developed a BAR-RPA assay to authenticate Traditional Chinese Medicine (TCM) products containing Wu Zhi Mao Tao, the roots of *Ficus hirta* Vahl. a valuable medicine and food ingredient found in China and effectively identified *Gelsemium elegans* (Gardner and Chapm.) Benth, a neurotoxic species often used as an adulterant in Wu Zhi Mao Tao.

### Microfluidics-based methods

Microfluidics, also known as a “laboratory on a chip,” is an advanced system that manipulates small amounts of fluid flowing in microscale channels with multidisciplinary applications (Whitesides, 2006). This system can integrate complex nucleic acid detection processes on a single chip with reduced operational time, low-cost consumption of samples and reagents, high capacity for multiplexing assays, and portability (Chen et al., 2022; Gao et al., 2022; Li et al., 2023). A variety of microfluidic devices have been developed for the rapid and efficient detection of foodborne pathogens, allergens, toxins, heavy metals, pesticide residues, additives, and other chemical and physical contaminants (Weng and Neethirajan, 2017; Chandrasekaran et al., 2022; Li et al., 2023). While recent research advances and typical microfluidic chip technologies have been summarized in Li et al. (2023), Jia et al. (2022) discussed the potential opportunities that microfluidic technology offers for pharmaceutical analysis, such as drug quality control, drug screening, and precision medicine.

### Microfluidics and loop-mediated isothermal amplification (microfluidics-based LAMP)

Fang et al. (2010) introduced the microLAMP (μLAMP) system, integrating nucleic acid LAMP into an 8-channel microfluidic chip, enabling the quantitative detection of pathogens in just an hour using a low amount of extracted DNA. Similarly, Sun et al. (2015) developed an 8-chamber lab-on-chip system that can detect and quantify *Salmonella* spp. in food samples. This platform performs on-chip sample preparation using magnetic beads and loop-mediated isothermal amplification (LAMP) for bacterial detection and can analyze eight Salmonella-spiked buffered peptone water (BPW) enriched pork meat samples within 40 min with a low Limit Of Detection (LOD) of 50 cells per test. Yuan et al. (2018) developed a colorimetric LAMP microfluidic chip-based method for detecting the allergen genes of *Arachis hypogaea* L. (peanut), *Sesamum indicum* L. (sesame), and *Glycine max* (L.) Merr. (soybean). The method has been successfully tested for various commercial foods (biscuits and candies), but could also be transferred to testing herbal products and dietary supplements.

### Clustered regularly interspaced short palindromic repeats (CRISPR)-microfluidic array

The development of Clustered Regularly Interspaced Short Palindromic Repeats (CRISPR) based biosensors has revolutionized the rapid detection of nucleic acids, allowing for faster diagnosis of infectious pathogens (Kellner et al., 2019; Li et al., 2021a) and identification of DNA or miRNAs from cancer cells (Li et al., 2021b). These biosensors have been further advanced through their integration with microfluidic platforms (Chen et al., 2022), with recent progress in this area reviewed by Li et al. (2022). Notably, Li et al. (2022) successfully developed a CRISPR-microfluidic array that enabled the identification of single-copy DNA mini barcodes, allowing for highly sensitive and specific discrimination of closely related *Datura* (Solanaceae) species.

### Microfluidic enrichment barcoding (MEBarcoding)

Gostel et al. (2020) proposed the Microfluidic Enrichment Barcoding (MEBarcoding) technique for plant high-throughput DNA barcoding. This innovative approach allows for the amplification of 48 DNA samples and hundreds of PCR primer pairs in a single thermal cycling protocol, using the Fluidigm Access Array. In their study, the authors successfully tested 96 samples using *rbcL*, *matK*, *trnH-psbA*, and ITS1 and 2 as barcode loci. The authors emphasized that MEBarcoding provides an efficient and viable alternative to traditional PCR and Sanger sequencing, particularly when a new barcode marker needs to be used to rapidly construct a customized reference library.

### Genome skimming (shallow pass shotgun sequencing)

Genome skimming is a technique that utilizes high-throughput sequencing (HTS) to sequence the genome at low coverage for the assembly of organellar genomes and nuclear ribosomal DNA sequences (Straub et al., 2012), allowing the capture of commonly used DNA barcoding markers (Coissac et al., 2016; Hollingsworth et al., 2016). High-throughput sequencing with the purpose of genome assembly requires high quality DNA, but for several genome skimming approaches, lower-quality DNA will suffice (Dodsworth, 2015). Such skimming approaches are sufficient to extract and assemble organelles genomes or identify species through k-mer analysis. Genome skimming has been successful in producing the entire nuclear genome of a 43-year-old *Arabidopsis thaliana* (L.) Heynh. herbarium specimen and the nuclear genome sequence of 80-year-old fungi (Staats et al., 2013), and recovered rDNA and plastid genome sequences of 80-year-old herbarium specimen using low concentrations of degraded material (Zeng et al., 2018). It has also been used in herbal product authentication to overcome the limitations of standard DNA barcoding and metabarcoding (Wu and Shaw, 2022). For instance, (Handy et al., 2021), compared the performance of DNA metabarcoding, genome skimming, and HPLC-UV analysis to authenticate 20 commercially available dietary supplements containing *Echinacea*. They found that genome skimming was more effective than DNA metabarcoding for species-level authentication within the *Echinacea* genus. However, the high operational cost of genome skimming currently limits its application (Manzanilla et al., 2022). Despite this limitation, the PCR-free approach of genome skimming bypasses some of the constraints of conventional barcoding, particularly concerning the limited number of barcodes used and the degraded DNA often present in herbal products (Wu and Shaw, 2022). For instance, Xin et al. (2018) performed shotgun sequencing of the Chinese herbal medicine Longdan Xiegan Wan and authenticated the raw material and products using ITS2, *psbA-trnH*, and *matK* sequences.

### Target capture sequencing

Target capture sequencing is a cost-effective alternative for genome subsampling, enabling high-throughput sequencing (HTS) of preselected nuclear loci (Gnirke et al., 2009; Andermann et al., 2019). This method has been successfully used to retrieve hundreds of genes from highly degraded DNA in herbarium specimens (Hart et al., 2016; Brewer et al., 2019) and in plant clades where traditional DNA barcoding has limited resolution (Widhelm et al., 2019; Woudstra et al., 2021). Universal target capture tools have improved the phylogenomic resolution at the species level of several plants (Johnson et al., 2019; McDonnell et al., 2021). For example, Manzanilla et al. (2022) used target capture genomic barcoding to identify and establish the geographic origin of the medicinal plant species, *Anacyclus pyrethrum* (L.) Lag., listed as Vulnerable on the IUCN Red List of Threatened Species. Although both target capture and genome skimming of plastomes resulted in similar numbers of reads per sample, target capture outperformed the latter. Despite its efficacy in handling low-quality DNA samples, the cost of target capture sequencing impedes its routine application for identifying commercialized plant species, as discussed for genome skimming. However, Woudstra et al. (2021) designed an RNA-bait panel targeting 189 low-copy nuclear genes that allows for accurate molecular identification of species from the genus *Aloe* L. This is particularly useful for the conservation and sustainable use of *Aloe*-derived products, as many *Aloe* species are regulated by the Convention on International Trade of Endangered Species (CITES). Additionally, Le et al. (2022) captured 353 nuclear markers using 319 individuals to evaluate the genetic diversity within cultivated and wild populations of *Panax vietnamensis* Ha and Grushv, an endemic and threatened ginseng species in Vietnam.

### MinION-based DNA metabarcoding

The MinION DNA sequencer, a portable device from Oxford Nanopore Technologies (ONT), has the ability to sequence individual DNA molecules as they pass through biological nanopores under an applied electrical field (Loman and Watson, 2015). Although the high error rate of MinION is currently a limiting factor, it is a promising technology for fast detection that can significantly reduce sequencing costs. To date, there are no reports on the use of MinION for herbal product authentication. However, Voorhuijzen-Harink et al. (2019) developed and evaluated a MinION-based DNA metabarcoding protocol using full-length DNA barcodes *cytb* and *COI* to identify fish species in two experimental mixtures. The performance was compared to Illumina MiSeq sequencing, and both technologies achieved the correct identification of all expected species with no false positive detections.

## Discussion

Over the last two decades, DNA-based methods have made a significant contribution to the quality control of the commercialization chain of herbal products. Early studies demonstrated the potential of the method and highlighted issues related to adulteration as well as the sensitivity of the technique (de Boer et al., 2015; Parveen et al., 2016; Sgamma et al., 2017; Ichim, 2019). The advent of DNA metabarcoding has provided additional tools for authenticating taxa in herbal products and assessing species diversity in complex herbals (Coghlan et al., 2015; Ivanova et al., 2016; Raclariu et al., 2017; Raclariu et al., 2018; Seethapathy et al., 2019). However, both DNA barcoding and DNA metabarcoding have limitations (Raclariu et al., 2018). One of these is the inability to detect negatives, which makes it challenging to determine whether an ingredient is absent or its DNA is undetectable (Novak et al., 2007; Costa et al., 2015; Raclariu et al., 2018). Additionally, false positives can occur, resulting in the detection of species that might be present in trace amounts in the production process or contaminations from environmental DNA (Ficetola et al., 2015). Recent research in DNA metabarcoding has focused on developing methods for quantifying herbal product ingredients, yielding meaningful results in a regulatory framework. Techniques, such as LAMP, BAR-RPA, and Bar-HRM, have made DNA-based methods more affordable as they require less costly equipment. Calibrated approaches of Bar-HRM also enable relative quantification of ingredients (Kalivas et al., 2014; Song et al., 2016; Osathanunkul and Madesis, 2019; Tungphatthong et al., 2021). Similarly, microfluidics-based methods offer tailored tests for the detection of target species at a potentially low cost (Li et al., 2023). Genomic barcoding approaches, such as target capture sequencing and genome skimming, are increasing the species identification resolution of DNA-based methods, enabling the distinction at an unprecedented scale of closely related species and even populations (Handy et al., 2021; Woudstra et al., 2021; Le et al., 2022; Manzanilla et al., 2022). Although many of these technologies are still at a proof-of-concept stage, it is challenging to predict which methods will find a role in routine quality control applications. One of the most promising of these is perhaps ddPCR, which enables the absolute quantification of DNA in samples and could be used to calibrate and quantify the amount of DNA from both intended and unwanted ingredients (Taylor et al., 2017; Xu et al., 2022; Yu et al., 2022). It is important to bear in mind that most of these tools yield specific and complementary data. Finding the right tool or tools to answer the specific research, monitoring or regulatory question at hand is key to harnessing the power of this expanding toolbox of DNA-based molecular methods.

## References

[B1] Abraham E. J. Kellogg J. J. (2021). Chemometric-guided approaches for profiling and authenticating botanical materials. Front. Nutr. 8, 780228. 10.3389/fnut.2021.780228 34901127 PMC8663772

[B2] Allgöwer S. M. Hartmann C. A. Lipinski C. Mahler V. Randow S. Völker E. (2020). LAMP-LFD based on isothermal amplification of multicopy gene ORF160b: Applicability for highly sensitive low-tech screening of allergenic soybean (*Glycine max*) in food. Foods 9 (12), 1741. 10.3390/foods9121741 33255927 PMC7760099

[B3] Alostad A. H. Steinke D. T. Schafheutle E. I. (2020). Herbal medicine classification: Policy recommendations. Front. Med. 7, 31. 10.3389/fmed.2020.00031 PMC7026670 32118016

[B4] Andermann T. Torres Jiménez M. F. Matos-Maraví P. Batista R. Blanco-Pastor J. L. Gustafsson A. L. S. (2019). A guide to carrying out a phylogenomic target sequence capture project. Front. Genet. 10, 1407. 10.3389/fgene.2019.01407 32153629 PMC7047930

[B5] Anthoons B. Karamichali I. Schrøder-Nielsen A. Drouzas A. D. de Boer H. Madesis P. (2021). Metabarcoding reveals low fidelity and presence of toxic species in short chain-of-commercialization of herbal products. J. Food Compos. Analysis 97, 103767. 10.1016/j.jfca.2020.103767

[B6] Arulandhu A. J. Staats M. Hagelaar R. Voorhuijzen M. M. Prins T. W. Scholtens I. (2017). Development and validation of a multi-locus DNA metabarcoding method to identify endangered species in complex samples. GigaScience 6 (10), 1–18. 10.1093/gigascience/gix080 PMC5632295 29020743

[B7] Baker D. A. Stevenson D. W. Little D. P. (2012). DNA barcode identification of black cohosh herbal dietary supplements. J. AOAC Int. 95 (4), 1023–1034. 10.5740/jaoacint.11-261 22970567

[B8] Booker A. Johnston D. Heinrich M. (2012). Value chains of herbal medicines--research needs and key challenges in the context of ethnopharmacology. J. Ethnopharmacol. 140 (3), 624–633. 10.1016/j.jep.2012.01.039 22326378

[B9] Brewer G. E. Clarkson J. J. Maurin O. Zuntini A. R. Barber V. Bellot S. (2019). Factors affecting targeted sequencing of 353 nuclear genes from herbarium specimens spanning the diversity of angiosperms. Front. Plant Sci. 10, 1102. 10.3389/fpls.2019.01102 31620145 PMC6759688

[B10] Chandrasekaran S. S. Agrawal S. Fanton A. Jangid A. R. Charrez B. Escajeda A. M. (2022). Rapid detection of SARS-CoV-2 RNA in saliva via Cas13. Nat. Biomed. Eng. 6 (8), 944–956. 10.1038/s41551-022-00917-y 35953650 PMC10367768

[B11] Chaudhary A. A. Khan M. Waleed M. A. S. Mohammad A. Osama A. A. K. (2014). Rapid and easy molecular authentication of medicinal plant *Zingiber officinale* roscoe by loop-mediated isothermal amplification (lamp)-based marker. J. Med. Plant Res. 8 (20), 756–762. 10.5897/JMPR2013.5351

[B12] Chaudhary A. A. H. Mohsin M. Ahmad A. (2012). Application of loop-mediated isothermal amplification (LAMP)-based technology for authentication of *Catharanthus roseus* (L) G Don. Protoplasma 249 (2), 417–422. 10.1007/s00709-011-0293-2 21644004

[B13] Chen B. Li Y. Xu F. Yang X. (2022). Powerful CRISPR-based biosensing techniques and their integration with microfluidic platforms. Front. Bioeng. Biotechnol. 10, 851712. 10.3389/fbioe.2022.851712 35284406 PMC8905290

[B14] Chen S. Yao H. Han J. Liu C. Song J. Shi L. (2010). Validation of the ITS2 region as a novel DNA barcode for identifying medicinal plant species. PLoS One 5, e8613–e8618. 10.1371/journal.pone.0008613 20062805 PMC2799520

[B15] Chen S. Sun Y. Fan F. Chen S. Zhang Y. Zhang Y. (2022). Present status of microfluidic PCR chip in nucleic acid detection and future perspective. TrAC Trends Anal. Chem. 157, 116737. 10.1016/j.trac.2022.116737

[B16] Cheng X. Su X. Chen X. Zhao H. Bo C. Xu J. (2014). Biological ingredient analysis of traditional Chinese medicine preparation based on high-throughput sequencing: The story for liuwei dihuang wan. Sci. Rep. 4, 5147. 10.1038/srep05147 24888649 PMC4042125

[B17] Coghlan M. L. Haile J. Houston J. Murray D. C. White N. E. Moolhuijzen P. (2012). Deep sequencing of plant and animal DNA contained within traditional Chinese medicines reveals legality issues and health safety concerns. PLoS Genet. 8 (4), e1002657. 10.1371/journal.pgen.1002657 22511890 PMC3325194

[B18] Coghlan M. L. Maker G. Crighton E. Haile J. Murray D. C. White N. E. (2015). Combined DNA, toxicological and heavy metal analyses provides an auditing toolkit to improve pharmacovigilance of traditional Chinese medicine (TCM). Sci. Rep. 5, 17475. 10.1038/srep17475 26658160 PMC4675079

[B19] Coissac E. Hollingsworth P. M. Lavergne S. Taberlet P. (2016). From barcodes to genomes: Extending the concept of DNA barcoding. Mol. Ecol. 25 (7), 1423–1428. 10.1111/mec.13549 26821259

[B20] Costa J. Amaral J. S. Fernandes T. J. R. Batista A. Oliveira M. B. P. P. Mafra I. (2015). DNA extraction from plant food supplements: Influence of different pharmaceutical excipients. Mol. Cell. Probes 29 (6), 473–478. 10.1016/j.mcp.2015.06.002 26079045

[B21] Craw P. Balachandran W. (2012). Isothermal nucleic acid amplification technologies for point-of-care diagnostics: A critical review. Lab A Chip 12 (14), 2469–2486. 10.1039/c2lc40100b 22592150

[B22] De Boer H. J. Ichim M. C. Newmaster S. G. (2015). DNA barcoding and pharmacovigilance of herbal medicines. Drug Saf. 38 (7), 611–620. 10.1007/s40264-015-0306-8 26076652

[B23] De Boer H. J. Hugo J. Ghorbani A. Manzanilla V. Raclariu A.-C. Kreziou A. (2017). DNA metabarcoding of orchid-derived products reveals widespread illegal orchid trade. Proc. Biol. Sci./ R. Soc. 284 (1863), 20171182. 10.1098/rspb.2017.1182 PMC5627200 28931735

[B24] Dodsworth S. (2015). Genome skimming for next-generation biodiversity analysis. Trends Plant Sci. 20 (9), 525–527. 10.1016/j.tplants.2015.06.012 26205170

[B25] Durazzo A. Sorkin B. C. Lucarini M. Gusev P. A. Kuszak A. J. Crawford C. (2021). Analytical challenges and metrological approaches to ensuring dietary supplement quality: International perspectives. Front. Pharmacol. 12, 714434. 10.3389/fphar.2021.714434 35087401 PMC8787362

[B26] Ekor M. (2014). The growing use of herbal medicines: Issues relating to adverse reactions and challenges in monitoring safety. Front. Pharmacol. 4, 177. 10.3389/fphar.2013.00177 24454289 PMC3887317

[B27] Fang X. Liu Y. Kong J. Jiang X. (2010). Loop-mediated isothermal amplification integrated on microfluidic chips for point-of-care quantitative detection of pathogens. Anal. Chem. 82 (7), 3002–3006. 10.1021/ac1000652 20218572

[B28] Ficetola G. F. Pansu J. Bonin A. Coissac E. Giguet-Covex C. De Barba M. (2015). Replication levels, false presences and the estimation of the presence/absence from eDNA metabarcoding data. Mol. Ecol. Resour. 15 (3), 543–556. 10.1111/1755-0998.12338 25327646

[B29] Fitzgerald M. Heinrich M. Booker A. (2019). Medicinal plant analysis: A historical and regional discussion of emergent complex techniques. Front. Pharmacol. 10, 1480. 10.3389/fphar.2019.01480 31998121 PMC6962180

[B30] Floren C. Wiedemann I. Brenig B. Schütz E. Beck J. (2015). Species identification and quantification in meat and meat products using droplet digital PCR (ddPCR). Food Chem. 173, 1054–1058. 10.1016/j.foodchem.2014.10.138 25466124

[B31] Fochi V. Sillo F. Travaglia F. Coïsson J. D. Balestrini R. Arlorio M. (2022). A Rapid and efficient loop-mediated isothermal amplification (LAMP) assay for the authentication of food supplements based on Maitake (*Grifola frondosa*). Food Anal. Methods 15 (7), 1803–1815. 10.1007/s12161-022-02235-0 35282313 PMC8903311

[B32] Frigerio J. Agostinetto G. Mezzasalma V. De Mattia F. Labra M. Bruno A. (2021). DNA-based herbal teas’ authentication: An ITS2 and psbA-trnH multi-marker DNA metabarcoding approach. Plants 10 (10), 2120. 10.3390/plants10102120 34685929 PMC8539046

[B33] Gafner S. Blumenthal M. Foster S. Cardellina J. H. Khan I. A. Upton R. (2023). Botanical ingredient forensics: Detection of attempts to deceive commonly used analytical methods for authenticating herbal dietary and food ingredients and supplements. J. Nat. Prod. 86, 460–472. 10.1021/acs.jnatprod.2c00929 36716213 PMC9972475

[B34] Galimberti A. De Mattia F. Losa A. Bruni I. Federici S. Casiraghi M. (2013). DNA barcoding as a new tool for food traceability. Food Res. Int. 50 (1), 55–63. 10.1016/j.foodres.2012.09.036

[B35] Gao D. Ma Z. Jiang Y. (2022). Recent advances in microfluidic devices for foodborne pathogens detection. TrAC Trends Anal. Chem. 157, 116788. 10.1016/j.trac.2022.116788

[B36] Gao Z. Liu Y. Wang X. Wei X. Han J. (2019). DNA mini-barcoding: A derived barcoding method for herbal molecular identification. Front. Plant Sci. 10, 987. 10.3389/fpls.2019.00987 31555305 PMC6724574

[B37] Garcia-Alvarez A. Egan B. de Klein S. Dima L. Maggi F. M. Isoniemi M. (2014). Usage of plant food supplements across six European countries: Findings from the PlantLIBRA consumer survey. Plos One 9 (3), e92265. 10.1371/journal.pone.0092265 24642692 PMC3958487

[B38] Ghorbani A. Saeedi Y. de Boer H. J. (2015). DNA barcoding in ethnobotany and ethnopharmacology: Identifying medicinal plants traded in local markets. Genome 58, 220.

[B39] Giguet-Covex C. Pansu J. Arnaud F. Rey P.-J. Griggo C. Gielly L. (2014). Long livestock farming history and human landscape shaping revealed by lake sediment DNA. Nat. Commun. 5, 3211. 10.1038/ncomms4211 24487920

[B40] Gnirke A. Melnikov A. Maguire J. Rogov P. LeProust E. M. Brockman W. (2009). Solution hybrid selection with ultra-long oligonucleotides for massively parallel targeted sequencing. Nat. Biotechnol. 27 (2), 182–189. 10.1038/nbt.1523 19182786 PMC2663421

[B41] Gostel M. R. Zúñiga J. D. Kress W. J. Funk V. A. Puente-Lelievre C. (2020). Microfluidic enrichment barcoding (MEBarcoding): A new method for high throughput plant DNA barcoding. Sci. Rep. 10 (1), 8701. 10.1038/s41598-020-64919-z 32457375 PMC7250904

[B42] Ha W.-Y. Wong K.-L. Ma W.-Y. Lau Y.-Y. Chan W.-H. (2022). Enhancing testing laboratory engagement in plant DNA barcoding through a routine workflow—A case study on Chinese materia medica (CMM). Plants 11, 1317. 10.3390/plants11101317 35631742 PMC9146924

[B43] Handy S. M. Pawar R. S. Ottesen A. R. Ramachandran P. Sagi S. Zhang N. (2021). HPLC-UV, metabarcoding and genome skims of botanical dietary supplements: A case study in *Echinacea* . Planta Medica 87 (4), 314–324. 10.1055/a-1336-1685 33445185

[B44] Hart M. L. Forrest L. L. Nicholls J. A. Kidner C. A. (2016). Retrieval of hundreds of nuclear loci from herbarium specimens. Taxon 65 (5), 1081–1092. 10.12705/655.9

[B45] Hawkins J. de Vere N. Griffith A. Ford C. R. Allainguillaume J. Hegarty M. J. (2015). Using DNA metabarcoding to identify the floral composition of honey: A new tool for investigating honey bee foraging preferences. Plos One 10 (8), e0134735. 10.1371/journal.pone.0134735 26308362 PMC4550469

[B46] Hebert P. D. N. Cywinska A. Ball S. L. deWaard J. R. (2003). Biological identifications through DNA barcodes. Proc. R. Soc. B/ Biol. Sci. 270 (1512), 313–321. 10.1098/rspb.2002.2218 PMC1691236 12614582

[B47] Heinrich M. Jalil B. Abdel-Tawab M. Echeverria J. Kulić Ž. McGaw L. J. (2022). Best Practice in the chemical characterisation of extracts used in pharmacological and toxicological research - the ConPhyMP-guidelines. Front. Pharmacol. 13, 953205. 10.3389/fphar.2022.953205 36176427 PMC9514875

[B48] Heinrich M. (2015). Quality and safety of herbal medical products: Regulation and the need for quality assurance along the value chains. Br. J. Clin. Pharmacol. 80 (1), 62–66. 10.1111/bcp.12586 25581270 PMC4500325

[B49] Heinrich M. Scotti F. Booker A. Fitzgerald M. Kum K. Y. Löbel K. (2019). Unblocking high-value botanical value chains: Is there a role for blockchain systems? Front. Pharmacol. 10, 396. 10.3389/fphar.2019.00396 31068810 PMC6491748

[B50] Hindson B. J. Ness K. D. Masquelier D. A. Belgrader P. Heredia N. J. Makarewicz A. J. (2011). High-throughput droplet digital PCR system for absolute quantitation of DNA copy number. Anal. Chem. 83 (22), 8604–8610. 10.1021/ac202028g 22035192 PMC3216358

[B51] Hindson C. M. Chevillet J. R. Briggs H. A. Gallichotte E. N. Ruf I. K. Hindson B. J. (2013). Absolute quantification by droplet digital PCR versus analog real-time PCR. Nat. Methods 10 (10), 1003–1005. 10.1038/nmeth.2633 23995387 PMC4118677

[B52] Hollingsworth P. M. Li D.-Z. van der Bank M. Twyford A. D. (2016). Telling plant species apart with DNA: From barcodes to genomes. Philosophical Trans. R. Soc. Lond. Ser. B, Biol. Sci. 371, 20150338. 10.1098/rstb.2015.0338 PMC4971190 27481790

[B53] Howard C. Bremner P. D. Fowler M. R. Isodo B. Scott N. W. Slater A. (2009). Molecular identification of *Hypericum perforatum* by PCR amplification of the ITS and 5.8S rDNA region. Planta Med. Jun 75 (8), 864–869. 10.1055/s-0029-1185397 19263343

[B54] Howard C. Hill E. Kreuzer M. Mali P. Masiero E. Slater A. (2020b). DNA authentication of st john's wort (*Hypericum perforatum* L) commercial products targeting the ITS region. Genes (Basel) 10 (4), 286. 10.3390/genes10040286 PMC6523358 30970623

[B55] Howard C. Lockie-Williams C. Slater A. (2020a). Applied barcoding: The practicalities of DNA testing for herbals. Plants (Basel) 9 (9), 1150. 10.3390/plants9091150 32899738 PMC7570336

[B56] Ichim M. C. Booker A. (2021). Chemical authentication of botanical ingredients: A review of commercial herbal products. Front. Pharmacol. 12, 666850. 10.3389/fphar.2021.666850 33935790 PMC8082499

[B57] Ichim M. C. Häser A. Nick P. (2020). Microscopic authentication of commercial herbal products in the globalized market: Potential and limitations. Front. Pharmacol. 11, 876. 10.3389/fphar.2020.00876 32581819 PMC7295937

[B58] Ichim M. C. (2019). The DNA-based authentication of commercial herbal products reveals their globally widespread adulteration. Front. Pharmacol. 10, 1227. 10.3389/fphar.2019.01227 31708772 PMC6822544

[B59] Ivanova N. V. Kuzmina M. L. Braukmann T. W. A. Borisenko A. V. Zakharov E. V. (2016). Authentication of herbal supplements using next-generation sequencing. Plos One 11 (5), e0156426. 10.1371/journal.pone.0156426 27227830 PMC4882080

[B60] Jaakola L. Suokas M. Häggman H. (2010). Novel approaches based on DNA barcoding and high-resolution melting of amplicons for authenticity analyses of berry species. Food Chem. 123 (2), 494–500. 10.1016/j.foodchem.2010.04.069

[B61] Jia X. Yang X. Luo G. Liang Q. (2022). Recent progress of microfluidic technology for pharmaceutical analysis. J. Pharm. Biomed. Analysis 209, 114534. 10.1016/j.jpba.2021.114534 34929566

[B62] Johnson M. G. Pokorny L. Dodsworth S. Botigué L. R. Cowan R. S. Devault A. (2019). A universal probe set for targeted sequencing of 353 nuclear genes from any flowering plant designed using k-medoids clustering. Syst. Biol. 68 (4), 594–606. 10.1093/sysbio/syy086 30535394 PMC6568016

[B63] Jordan C. R. Harris C. M. Miranda M. I. Kim D. Y. Hellberg R. S. (2023). Labeling compliance and online claims for Ayurvedic herbal supplements on the U.S. market associated with the purported treatment of COVID-19. Food control. 148, 109673. 10.1016/j.foodcont.2023.109673 36778101 PMC9901855

[B64] Kalivas A. Ganopoulos I. Xanthopoulou A. Chatzopoulou P. Tsaftaris A. Madesis P. (2014). DNA barcode ITS2 coupled with high resolution melting (HRM) analysis for taxonomic identification of Sideritis species growing in Greece. Mol. Biol. Rep. 41 (8), 5147–5155. 10.1007/s11033-014-3381-5 24802796

[B65] Kellner M. J. Koob J. G. Gootenberg J. S. Abudayyeh O. O. Zhang F. (2019). Sherlock: Nucleic acid detection with CRISPR nucleases. Nat. Protoc. 14 (10), 2986–3012. 10.1038/s41596-019-0210-2 31548639 PMC6956564

[B66] Klein-Junior L. C. de Souza M. R. Viaene J. Bresolin T. M. B. de Gasper A. L. Henriques A. T. (2021). Quality control of herbal medicines: From traditional techniques to state-of-the-art approaches. Planta Medica 87 (12–13), 964–988. 10.1055/a-1529-8339 34412146

[B67] Kogovšek P. Hodgetts J. Hall J. Prezelj N. Nikolić P. Mehle N. (2015). LAMP assay and rapid sample preparation method for on-site detection of flavescence dorée phytoplasma in grapevine. Plant Pathol. 64 (2), 286–296. 10.1111/ppa.12266 26146413 PMC4480326

[B68] Köppel R. Ganeshan A. Weber S. Pietsch K. Graf C. Hochegger R. (2019). Duplex digital PCR for the determination of meat proportions of sausages containing meat from chicken, Turkey, horse, cow, pig and sheep. Eur. Food Res. Technol. 245 (4), 853–862. 10.1007/s00217-018-3220-3

[B69] Kreuzer M. Howard C. Adhikari B. Pendry C. A. Hawkins J. A. (2019). Phylogenomic approaches to DNA barcoding of herbal medicines: Developing clade-specific diagnostic characters for *berberis* . Front. Plant Sci. 10, 586. 10.3389/fpls.2019.00586 31139202 PMC6527895

[B70] Lai G.-H. Chao J. Lin M.-K. Chang W.-T. Peng W.-H. Sun F.-C. (2015). Rapid and sensitive identification of the herbal tea ingredient *Taraxacum formosanum* using loop-mediated isothermal amplification. Int. J. Mol. Sci. 16 (1), 1562–1575. 10.3390/ijms16011562 25584616 PMC4307320

[B71] Law J. W.-F. Ab Mutalib N.-S. Chan K.-G. Lee L.-H. (2014). Rapid methods for the detection of foodborne bacterial pathogens: Principles, applications, advantages and limitations. Front. Microbiol. 5, 770. 10.3389/fmicb.2014.00770 25628612 PMC4290631

[B72] Le H. T. T. Nguyen L. N. Pham H. L. B. Le H. T. M. Luong T. D. Huynh H. T. T. (2022). Target capture reveals the complex origin of Vietnamese ginseng. Front. Plant Sci. 13, 814178. 10.3389/fpls.2022.814178 35909770 PMC9326450

[B73] Li B. Shao Z. Chen Y. (2021a). An exonuclease protection and CRISPR/Cas12a integrated biosensor for the turn-on detection of transcription factors in cancer cells. Anal. Chim. Acta 1165, 338478. 10.1016/j.aca.2021.338478 33975701

[B74] Li B. Ye Q. Chen M. Zhou B. Zhang J. Pang R. (2021b). An ultrasensitive CRISPR/Cas12a based electrochemical biosensor for *Listeria monocytogenes* detection. Biosens. Bioelectron. 179, 113073. 10.1016/j.bios.2021.113073 33581428

[B75] Li J.-J. Xiong C. Liu Y. Liang J.-S. Zhou X.-W. (2016). Loop-mediated isothermal amplification (LAMP): Emergence as an alternative technology for herbal medicine identification. Front. Plant Sci. 7, 1956. 10.3389/fpls.2016.01956 28082999 PMC5183589

[B76] Li M. Wong Y.-L. Jiang L.-L. Wong K.-L. Wong Y.-T. Lau C. B.-S. (2013). Application of novel loop-mediated isothermal amplification (LAMP) for rapid authentication of the herbal tea ingredient *Hedyotis diffusa* Willd. Food Chem. 141 (3), 2522–2525. 10.1016/j.foodchem.2013.05.085 23870990

[B77] Li P. Xiong H. Yang B. Jiang X. Kong J. Fang X. (2022). Recent progress in CRISPR-based microfluidic assays and applications. TrAC Trends Anal. Chem. 157, 116812. 10.1016/j.trac.2022.116812

[B78] Li Z. Xu X. Wang D. Jiang X. (2023). Recent advancements in nucleic acid detection with microfluidic chip for molecular diagnostics. Trends Anal. Chem. TRAC 158, 116871. 10.1016/j.trac.2022.116871 PMC9721164 36506265

[B79] Little D. P. (2014b). A DNA mini-barcode for land plants. Mol. Ecol. Resour. 14 (3), 437–446. 10.1111/1755-0998.12194 24286499

[B80] Little D. P. (2014a). Authentication of *Ginkgo biloba* herbal dietary supplements using DNA barcoding. Genome Natl. Res. Counc. Can. Génome Cons. Natl. Rech. Can. 57, 513–516. 10.1139/gen-2014-0130 25495290

[B81] Lobato I. M. O’Sullivan C. K. (2018). Recombinase polymerase amplification: Basics, applications and recent advances. Trends Anal. Chem. TRAC 98, 19–35. 10.1016/j.trac.2017.10.015 PMC7112910 32287544

[B82] Loman N. J. Watson M. (2015). Successful test launch for nanopore sequencing. Nat. Methods 12 (4), 303–304. 10.1038/nmeth.3327 25825834

[B83] Manzanilla V. Teixidor-Toneu I. Martin G. J. Hollingsworth P. M. de Boer H. J. Kool A. (2022). Using target capture to address conservation challenges: Population-level tracking of a globally-traded herbal medicine. Mol. Ecol. Resour. 22 (1), 212–224. 10.1111/1755-0998.13472 34270854

[B84] Mao R. Xie K. Zhao M. Li M. Lu L. Liu Y. (2020). Development and evaluation of a loop-mediated isothermal amplification (LAMP) assay for rapid detection of pistachio (*Pistacia vera*) in food samples. Food Anal. Methods 13 (3), 658–666. 10.1007/s12161-019-01684-4

[B85] McDonnell A. J. Baker W. J. Dodsworth S. Forest F. Graham S. W. Johnson M. G. (2021). Exploring Angiosperms353: Developing and applying a universal toolkit for flowering plant phylogenomics. Appl. Plant Sci. 9 (7), 11443. 10.1002/aps3.11443 PMC8312743 34336400

[B86] Meusnier I. Singer G. A. Landry J. F. Hickey D. A. Hebert P. D. Hajibabaei M. (2008). A universal DNA mini-barcode for biodiversity analysis. BMC Genomics 12 (9), 214. 10.1186/1471-2164-9-214 PMC2396642 18474098

[B87] Meyer C. P. Paulay G. (2005). DNA barcoding: Error rates based on comprehensive sampling. PLoS Biol. 3 (12), e422. 10.1371/journal.pbio.0030422 16336051 PMC1287506

[B88] Niessen L. Luo J. Denschlag C. Vogel R. F. (2013). The application of loop-mediated isothermal amplification (LAMP) in food testing for bacterial pathogens and fungal contaminants. Food Microbiol. 36 (2), 191–206. 10.1016/j.fm.2013.04.017 24010598

[B89] Notomi T. Okayama H. Masubuchi H. Yonekawa T. Watanabe K. Amino N. (2000). Loop-mediated isothermal amplification of DNA. Nucleic Acids Res. 28 (12), 63e–663e. 10.1093/nar/28.12.e63 PMC102748 10871386

[B90] Novak J. Grausgruber-Gröger S. Lukas B. (2007). DNA-based authentication of plant extracts. Food Res. Int. 40 (3), 388–392. 10.1016/j.foodres.2006.10.015

[B91] Osathanunkul M. Madesis P. (2019). Bar-HRM: A reliable and fast method for species identification of ginseng (*Panax ginseng, Panax notoginseng, Talinum paniculatum* and *Phytolacca americana*). PeerJ 7, e7660. 10.7717/peerj.7660 31579587 PMC6765363

[B92] Osathanunkul M. Madesis P. de Boer H. (2015). Bar-HRM for authentication of plant-based medicines: Evaluation of three medicinal products derived from *Acanthaceae* species. Plos One 10 (5), e0128476. 10.1371/journal.pone.0128476 26011474 PMC4444109

[B93] Parveen I. Gafner S. Techen N. Murch S. J. Khan I. A. (2016). DNA barcoding for the identification of botanicals in herbal medicine and dietary supplements: Strengths and limitations. Planta Medica 82 (14), 1225–1235. 10.1055/s-0042-111208 27392246

[B94] Piepenburg O. Williams C. H. Stemple D. L. Armes N. A. (2006). DNA detection using recombination proteins. PLoS Biol. 4 (7), e204. 10.1371/journal.pbio.0040204 16756388 PMC1475771

[B95] Pinheiro L. B. Coleman V. A. Hindson C. M. Herrmann J. Hindson B. J. Bhat S. (2012). Evaluation of a droplet digital polymerase chain reaction format for DNA copy number quantification. Anal. Chem. 84 (2), 1003–1011. 10.1021/ac202578x 22122760 PMC3260738

[B96] Piñol J. Mir G. Gomez-Polo P. Agustí N. (2015). Universal and blocking primer mismatches limit the use of high-throughput DNA sequencing for the quantitative metabarcoding of arthropods. Mol. Ecol. Resour. 15 (4), 819–830. 10.1111/1755-0998.12355 25454249

[B97] Polling M. Sin M. de Weger L. A. Speksnijder A. G. C. L. Koenders M. J. F. de Boer H. (2022). DNA metabarcoding using nrITS2 provides highly qualitative and quantitative results for airborne pollen monitoring. Sci. Total Environ. 806 (1), 150468. 10.1016/j.scitotenv.2021.150468 34583071 PMC8651626

[B98] Posadzki P. Watson L. K. Ernst E. (2013). Adverse effects of herbal medicines: An overview of systematic reviews. Clin. Med. 13 (1), 7–12. 10.7861/clinmedicine.13-1-7 PMC5873713 23472485

[B99] Raclariu A. C. Mocan A. Popa M. O. Vlase L. Ichim M. C. Crisan G. (2017). *Veronica officinalis* product authentication using DNA metabarcoding and HPLC-MS reveals widespread adulteration with *Veronica chamaedrys* . Front. Pharmacol. 8, 378. 10.3389/fphar.2017.00378 28674497 PMC5474480

[B100] Raclariu A. C. Heinrich M. Ichim M. C. de Boer H. (2018). Benefits and limitations of DNA barcoding and metabarcoding in herbal product authentication. Phytochem. Anal. 29 (2), 123–128. 10.1002/pca.2732 28906059 PMC5836936

[B101] Sasaki Y. Nagumo S. (2007). Rapid identification of Curcuma longa and C. aromatica by LAMP. Biol. Pharm. Bull. 30 (11), 2229–2230. 10.1248/bpb.30.2229 17978508

[B102] Seethapathy G. S. Ganesh D. Kumar J. U. S. Senthilkumar U. Newmaster S. G. Ragupathy S. (2014). Assessing product adulteration in natural health products for laxative yielding plants, *Cassia, Senna*, and *Chamaecrista*, in Southern India using DNA barcoding. Int. J. Leg. Med. 129, 693–700. 10.1007/s00414-014-1120-z 25425095

[B103] Seethapathy G. S. Raclariu-Manolica A.-C. Anmarkrud J. A. Wangensteen H. de Boer H. J. (2019). DNA metabarcoding authentication of ayurvedic herbal products on the European market raises concerns of quality and fidelity. Front. Plant Sci. 10, 68. 10.3389/fpls.2019.00068 30804961 PMC6370972

[B104] Sgamma T. Lockie-Williams C. Kreuzer M. Williams S. Scheyhing U. Koch E. (2017). DNA barcoding for industrial quality assurance. Planta Medica 83 (14–15), 1117–1129. 10.1055/s-0043-113448 28662530

[B105] Sgamma T. Masiero E. Mali P. Mahat M. Slater A. (2018). Sequence-specific detection of *Aristolochia* DNA – A simple test for contamination of herbal products. Front. Plant Sci. 9, 1828. 10.3389/fpls.2018.01828 30619401 PMC6297175

[B106] Sheu S.-C. Tsou P.-C. Lien Y.-Y. Lee M.-S. (2018). Development of loop-mediated isothermal amplification (LAMP) assays for the rapid detection of allergic peanut in processed food. Food Chem. 257, 67–74. 10.1016/j.foodchem.2018.02.124 29622231

[B107] Shipkowski K. A. Betz J. M. Birnbaum L. S. Bucher J. R. Coates P. M. Hopp D. C. (2018). Naturally complex: Perspectives and challenges associated with botanical dietary supplement safety assessment. Food Chem. Toxicol. 118, 963–971. 10.1016/j.fct.2018.04.007 29626579 PMC6087675

[B108] Sillo F. Giordano L. Gonthier P. (2018). Fast and specific detection of the invasive forest pathogen *Heterobasidion irregulare* through a Loop-mediated isothermal amplification (LAMP) assay. For. Pathol. 48 (2), e12396. 10.1111/efp.12396

[B109] Simmler C. Graham J. G. Chen S.-N. Pauli G. F. (2018). Integrated analytical assets aid botanical authenticity and adulteration management. Fitoterapia 129, 401–414. 10.1016/j.fitote.2017.11.017 29175549 PMC5963993

[B110] Singh M. Pal D. Sood P. Randhawa G. (2019). Loop-mediated isothermal amplification assays: Rapid and efficient diagnostics for genetically modified crops. Food control. 106, 106759. 10.1016/j.foodcont.2019.106759

[B111] Smith T. Majid F. Eckl V. Morton Reynolds C. (2021). Herbal supplement sales in US increase by record-breaking 17.3% in 2020. HerbalGram 131, 52–65.

[B112] Song M. Li J. Xiong C. Liu H. Liang J. (2016). Applying high-resolution melting (HRM) technology to identify five commonly used *Artemisia* species. Sci. Rep. 6, 34133. 10.1038/srep34133 27698485 PMC5048426

[B113] Speranskaya A. S. Khafizov K. Ayginin A. A. Krinitsina A. A. Omelchenko D. O. Nilova M. V. (2018). Comparative analysis of Illumina and Ion Torrent high-throughput sequencing platforms for identification of plant components in herbal teas. Food control. 93, 315–324. 10.1016/j.foodcont.2018.04.040

[B114] Staats M. Erkens R. H. J. van de Vossenberg B. Wieringa J. J. Kraaijeveld K. Stielow B. (2013). Genomic treasure troves: Complete genome sequencing of herbarium and insect museum specimens. Plos One 8 (7), e69189. 10.1371/journal.pone.0069189 23922691 PMC3726723

[B115] Steinhoff B. (2019). Pyrrolizidine alkaloid contamination in herbal medicinal products: Limits and occurrence. Food Chem. Toxicol. 130, 262–266. 10.1016/j.fct.2019.05.026 31121208

[B116] Straub S. C. K. Parks M. Weitemier K. Fishbein M. Cronn R. C. Liston A. (2012). Navigating the tip of the genomic iceberg: Next-generation sequencing for plant systematics. Am. J. Bot. 99 (2), 349–364. 10.3732/ajb.1100335 22174336

[B117] Sun W. Li J.-J. Xiong C. Zhao B. Chen S.-L. (2016). The potential power of Bar-HRM technology in herbal medicine identification. Front. Plant Sci. 7, 367. 10.3389/fpls.2016.00367 27066026 PMC4811891

[B118] Sun Y. Quyen T. L. Hung T. Q. Chin W. H. Wolff A. Bang D. D. (2015). A lab-on-a-chip system with integrated sample preparation and loop-mediated isothermal amplification for rapid and quantitative detection of *Salmonella* spp. in food samples. Lab A Chip 15 (8), 1898–1904. 10.1039/c4lc01459f 25715949

[B119] Taberlet P. Coissac E. Pompanon F. Brochmann C. Willerslev E. (2012). Towards next-generation biodiversity assessment using DNA metabarcoding. Mol. Ecol. 21 (8), 2045–2050. 10.1111/j.1365-294X.2012.05470.x 22486824

[B120] Taylor S. C. Laperriere G. Germain H. (2017). Droplet digital PCR versus qPCR for gene expression analysis with low abundant targets: From variable nonsense to publication quality data. Sci. Rep. 7 (1), 2409. 10.1038/s41598-017-02217-x 28546538 PMC5445070

[B121] Techen N. Parveen I. Pan Z. Khan I. A. (2014). DNA barcoding of medicinal plant material for identification. Curr. Opin. Biotechnol. 25, 103–110. 10.1016/j.copbio.2013.09.010 24484887

[B122] Teschke R. Eickhoff A. (2015). Herbal hepatotoxicity in traditional and modern medicine: Actual key issues and new encouraging steps. Front. Pharmacol. 6, 72. 10.3389/fphar.2015.00072 25954198 PMC4407580

[B123] Thakkar S. Anklam E. Xu A. Ulberth F. Li J. Li B. (2020). Regulatory landscape of dietary supplements and herbal medicines from a global perspective. Regul. Toxicol. Pharmacol. 114, 104647. 10.1016/j.yrtph.2020.104647 32305367

[B124] Tian E. Liu Q. Ye H. Li F. Chao Z. (2017). A DNA barcode-based RPA Assay (BAR-RPA) for rapid identification of the dry root of *Ficus hirta* (Wuzhimaotao). Mol. (Basel, Switz. 22 (12), 2261. 10.3390/molecules22122261 PMC6149672 29258287

[B125] Tungphatthong C. Urumarudappa S. K. J. Awachai S. Sooksawate T. Sukrong S. (2021). Differentiation of *Mitragyna speciosa*, a narcotic plant, from allied *Mitragyna* species using DNA barcoding-high-resolution melting (Bar-HRM) analysis. Sci. Rep. 11 (1), 6738. 10.1038/s41598-021-86228-9 33762644 PMC7990970

[B126] Upton R. David B. Gafner S. Glasl S. (2019). Botanical ingredient identification and quality assessment: Strengths and limitations of analytical techniques. Phytochemistry Rev. Proc. Phytochemical Soc. Eur. 19, 1157–1177. 10.1007/s11101-019-09625-z

[B127] Voorhuijzen-Harink M. M. Hagelaar R. van Dijk J. P. Prins T. W. Kok E. J. Staats M. (2019). Toward on-site food authentication using nanopore sequencing. Food Chem. X 2, 100035. 10.1016/j.fochx.2019.100035 31432019 PMC6694865

[B128] Wallace L. J. Boilard S. M. A. L. Eagle S. H. C. Spall J. L. Shokralla S. Hajibabaei M. (2012). DNA barcodes for everyday life: Routine authentication of natural health products. Food Res. Int. 49, 446–452. 10.1016/j.foodres.2012.07.048

[B129] Wang Q. Cai Y. He Y. Yang L. Li J. Pan L. (2017). Droplet digital PCR (ddPCR) method for the detection and quantification of goat and sheep derivatives in commercial meat products. Eur. Food Res. Technol. 244 (4), 767–774. 10.1007/s00217-017-3000-5

[B130] Weng X. Neethirajan S. (2017). Ensuring food safety: Quality monitoring using microfluidics. Trends Food Sci. Technol. 65, 10–22. 10.1016/j.tifs.2017.04.015

[B131] Whitesides G. M. (2006). The origins and the future of microfluidics. Nature 442 (7101), 368–373. 10.1038/nature05058 16871203

[B132] Widhelm T. J. Grewe F. Huang J.-P. Mercado-Díaz J. A. Goffinet B. Lücking R. (2019). Multiple historical processes obscure phylogenetic relationships in a taxonomically difficult group (*Lobariaceae*, Ascomycota). Sci. Rep. 9 (1), 8968. 10.1038/s41598-019-45455-x 31222061 PMC6586878

[B133] Woudstra Y. Viruel J. Fritzsche M. Bleazard T. Mate R. Howard C. (2021). A customised target capture sequencing tool for molecular identification of *Aloe vera* and relatives. Sci. Rep. 11 (1), 24347. 10.1038/s41598-021-03300-0 34934068 PMC8692607

[B134] Wu H.-Y. Shaw P.-C. (2022). Strategies for molecular authentication of herbal products: From experimental design to data analysis. Chin. Med. 17 (1), 38. 10.1186/s13020-022-00590-y 35317843 PMC8939074

[B135] Xin T. Su C. Lin Y. Wang S. Xu Z. Song J. (2018). Precise species detection of traditional Chinese patent medicine by shotgun metagenomic sequencing. Phytomedicine Int. J. Phytotherapy Phytopharm. 47, 40–47. 10.1016/j.phymed.2018.04.048 30166107

[B136] Xu W. Zhu P. Xin T. Lou Q. Li R. Fu W. (2022). Droplet digital PCR for the identification of plant-derived adulterants in highly processed products. Phytomedicine Int. J. Phytotherapy Phytopharm. 105, 154376. 10.1016/j.phymed.2022.154376 35963193

[B137] You H. Abraham E. J. Mulligan J. Zhou Y. Montoya M. Willig J. (2022). Label compliance for ingredient verification: Regulations, approaches, and trends for testing botanical products marketed for “immune health” in the United States. Crit. Rev. Food Sci. Nutr. 19, 1–20. 10.1080/10408398.2022.2124230 36123797

[B138] Yu N. Han J. Deng T. Chen L. Zhang J. Xing R. (2020). A novel analytical droplet digital PCR method for identification and quantification of raw health food material powder from *Panax notoginseng* . Food Anal. Methods 14, 552–560. 10.1007/s12161-020-01887-0

[B139] Yu N. Xing R. Wang P. Deng T. Zhang J. Zhao G. (2022). A novel duplex droplet digital PCR assay for simultaneous authentication and quantification of *Panax notoginseng* and its adulterants. Food control. 132, 108493. 10.1016/j.foodcont.2021.108493

[B140] Yu X. Tan W. Gao H. Miao L. Tian X. (2020). Development of a specific mini-barcode from plastome and its application for qualitative and quantitative identification of processed herbal products using DNA metabarcoding technique: A case study on *Senna* . Front. Pharmacol. 11, 585687. 10.3389/fphar.2020.585687 33390955 PMC7773718

[B141] Yuan D. Kong J. Li X. Fang X. Chen Q. (2018). Colorimetric LAMP microfluidic chip for detecting three allergens: Peanut, sesame and soybean. Sci. Rep. 8, 8682. 10.1038/s41598-018-26982-5 29875429 PMC5989197

[B142] Zeng C.-X. Hollingsworth P. M. Yang J. He Z.-S. Zhang Z.-R. Li D.-Z. (2018). Genome skimming herbarium specimens for DNA barcoding and phylogenomics. Plant Methods 14, 43. 10.1186/s13007-018-0300-0 29928291 PMC5987614

[B143] Zhang J. Wider B. Shang H. Li X. Ernst E. (2012). Quality of herbal medicines: Challenges and solutions. Complementary Ther. Med. 20 (1–2), 100–106. 10.1016/j.ctim.2011.09.004 22305255

[B144] Zhao M. Shi Y. Wu L. Guo L. Liu W. Xiong C. (2016). Rapid authentication of the precious herb saffron by loop-mediated isothermal amplification (LAMP) based on internal transcribed spacer 2 (ITS2) sequence. Sci. Rep. 6, 25370. 10.1038/srep25370 27146605 PMC4857077

[B145] Zhong J. Zhao X. (2018). Isothermal amplification technologies for the detection of foodborne pathogens. Food Anal. Methods 11 (6), 1543–1560. 10.1007/s12161-018-1177-2

[B146] Zuo Y. Chen Z. Kondo K. Funamoto T. Wen J. Zhou S. (2011). DNA barcoding of Panax species. Planta Med. 77, 182–187. 10.1055/s-0030-1250166 20803416

